# Lassa Fever Virus Binds Matriglycan—A Polymer of Alternating Xylose and Glucuronate—On α-Dystroglycan

**DOI:** 10.3390/v13091679

**Published:** 2021-08-25

**Authors:** Soumya Joseph, Kevin P. Campbell

**Affiliations:** Howard Hughes Medical Institute, Senator Paul D. Wellstone Muscular Dystrophy Specialized Research Center, Department of Molecular Physiology and Biophysics and Department of Neurology, Roy J. and Lucille A. Carver College of Medicine, The University of Iowa, Iowa City, IA 52242, USA; soumya-joseph@uiowa.edu

**Keywords:** Lassa fever virus, matriglycan, α-dystroglycan, lymphocytic choriomeningitis, Axl, Gas6, dystrophin-glycoprotein complex, LARGE1, laminin, apoptotic mimicry

## Abstract

Lassa fever virus (LASV) can cause life-threatening hemorrhagic fevers for which there are currently no vaccines or targeted treatments. The late Prof. Stefan Kunz, along with others, showed that the high-affinity host receptor for LASV, and other Old World and clade-C New World mammarenaviruses, is matriglycan—a linear repeating disaccharide of alternating xylose and glucuronic acid that is polymerized uniquely on α-dystroglycan by like-acetylglucosaminyltransferase-1 (LARGE1). Although α-dystroglycan is ubiquitously expressed, LASV preferentially infects vascular endothelia and professional phagocytic cells, which suggests that viral entry requires additional cell-specific factors. In this review, we highlight the work of Stefan Kunz detailing the molecular mechanism of LASV binding and discuss the requirements of receptors, such as tyrosine kinases, for internalization through apoptotic mimicry.

## 1. Introduction

**Figure 1:**
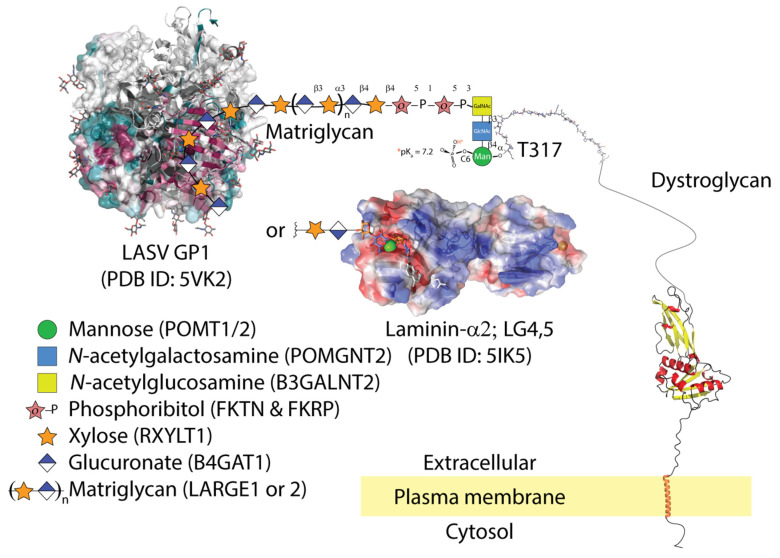
LASV GP1 is able to bind matriglycan (xylose and glucuronate), but only gains entry to cells that co-express apoptotic phagocytic machinery. The molecular details of LASV GP1 binding to matriglycan are unknown. Matriglycan is polymerized on a primer of extended phosphocore M3 on threonine-317 and possibly 379 of α-dystroglycan. The core M3 trisaccharide is phosphorylated by Protein *O*-Mannosyl Kinase (POMK); other glycosyltransferases are listed in parentheses next to their corresponding sugars. The conserved surface residues of LASV trimer from 5VK2 are shown as a gradient of magenta (conserved) to green (non-conserved); (accessed on 6 July 2021: https://consurf.tau.ac.il/). LASV GP1 binding displaces LG domains from matriglycan. The semi-transparent electrostatic surface of LG4-5 domains from laminin-2α is shown binding a unit of xylose-glucuronate via calcium (green sphere). Parts of the dystroglycan structure were downloaded from AlphaFold [26].

The surfaces of host cells and pathogens are covered with glycoproteins. Glycans are well-known targets for virus binding and entry [1]. We highlight the work of the late Stefan Kunz who showed the surface glycoprotein of Old World, including LASV, and New World clade-C mammarenaviruses, binds matriglycan [2,3,4,5]—a polysaccharide of alternating xylose and glucuronate—on the highly glycosylated mucin-like receptor, dystroglcyan on rodent and human hosts (Figure 1). On the other hand, the spike glycoproteins (GP) of New World mammarenavirus clades use transferrin as a receptor [6,7,8]. We limit the scope of our review to virus binding and entry. Other publications detailing events post cell-entry and broader aspects of arenaviruses have been published [9,10,11,12].

The high-affinty receptor for the trimeric glycoprotein-1 (GP1) of Old World and clade-C New World mammarenaviruses is matriglycan, which is polymerized on α-dystroglycan post-translationally by like-acetylglucosaminyltransferase (LARGE1) [13]. Dystroglycan consists of α-dystroglycan and β-dystroglycan [14] and is part of the plasma membrane-embedded dystrophin-glycoprotein-complex (DGC), which is ubiquitously expressed but whose composition varies between cell types [15,16,17]. Under normal circumstances, proteins that contain laminin-globular (LG) domains bind matriglycan and components of the DGC bind the actin cytoskeleton such that, at its simplest, the DGC anchors the cytoskeleton to the extracellular matrix (ECM) [18,19,20,21,22]. Genetic mutations that diminish or abolish matriglycan polymerization can cause muscular dystrophies, known as dystroglycanopathies, with or without brain and eye involvement [23,24]. Much like mutations that cause sickle-cell anemia can protect against malarial parasites, LARGE1 alleles have undergone recent positive selection in West African populations where LASV is endemic [25].

Despite ubiquitous expression of matriglycan on dystroglycan throughout human hosts, LASV preferentially infects antigen-presenting cells, including dendritic cells [27], but not muscle cells [28], which suggests that additional factors influence viral entry. The differential manifestations of both genetic and infectious diseases indicate that dystroglycan associates with dynamic, cell-type-specific complexes [29,30] on which pathologies depend. Many reports [31,32,33] have implicated auxiliary cell surface receptors as co-factors for viral entry: the tyrosine kinase family, Tyro3/Axl/MERTK (TAM) or hepatocyte growth factor receptor (HGFR) [34], T-cell immunoglobulin and mucin domain-containing proteins that bind phosphatidylserine, TIM1 and 4 [35], and the lectin family-4 members M and G (CLEC4G and M) that are popularly known as DC-SIGN and LSECtin [33].

LASV is a highly prevalent mammarenavirus in Western Africa, where it infects a few hundred thousand individuals annually [36]. Lassa fever (LF) is associated with high morbidity and a recent average case fatality rate of ~20% in Nigeria [37]. Currently, neither a vaccine nor specific treatments for LF exist. The majority of basic scientific knowledge on LASV entry was generated in the Oldstone laboratory (Scripps Reseach Institute) and much of the detailed analysis was carried out by the late Dr. Stefan Kunz, first as a post-doctoral fellow in the Oldstone laboratory and later as an independent investigator at the University of Lausanne.

Conveniently, lymphocytic choriomeningitis virus (LCMV) and LASV both use α-dystroglycan as a high-affinity receptor [2]. Results from virus overlay protein blot assays (VOPBA), which are like Western blots but use whole virus particles instead of primary antibodies, suggested that an SDS-resistant moiety on α-dystroglycan is responsible for binding virus particles. Additionally, viral infectivity was restored in α-dystroglycan-deficient cells by adenoviral dystroglycan transductions. Purified soluble α-dystroglycan blocked cell entry by acting as a decoy receptor. These initial studies also showed that bacterially expressed α-dystroglycan could not bind virus particles, suggesting that a mammalian-specific post-translational modification, perhaps glycosylation, was crucial to binding [2]. This formed the basis of Dr. Kunz’ invaluable work on mammarenaviral cell entry. Using a series of dystroglycan deletion mutants, he deduced that residues 169–408 of dystroglycan were essential for virus binding and that the viral glycoprotein bound the same post-translational modification as laminin [4]. This work culminated in the understanding that GP1 from LASV, LCMV, Mobala, and Oliveros mammarenavirus [5] bound a chain of alternating xylose and glucuronic acid that is polymerized by LARGE1 on α-dystroglycan residues threonine-317 [13,38]. His conclusions were bosltered by the fact that the overexpression of LARGE1 increased mammarenaviral binding [5,39]. Interestingly, a novel genetic screen using haploid cells confirmed these results [40]. Also, replacing the transmembrane and cytosolic domains of β-dystroglycan did not affect viral entry [41]. This, along with tissue tropism, implicates auxiliary signalling receptors in viral entry. This was the foundation of Dr. Kunz’ independent research.

## 2. LASV Binds Matriglycan and Is Internalized in Cells Co-Expressing Gas6-Axl

Phosphorylation of intracellular auxiliary receptors is key to LASV cell entry [31,32,42,43]. The application of the generic tyrosine kinase inhibitor genistein prevented virus internalization but not binding [34,42], which suggests that virus binding and internalization are separable. For example, tyrosine-892 on β -dystroglycan is phosphorylated in response to virus binding. The Tyro3/Axl/MERTK (TAM) tyrosine kinases that are expressed on dendritic cells can promote the internalization of virus particles via receptor phosphorylation [17,42].

Axl bound to its co-factor growth arrest-specific protein-6 (Gas6) is expressed in cells that phagocytose apoptotic cells and is key to internalizing other viruses such as Zika, Dengue, and Ebola [32,44,45,46], which use apoptotic mimicry [47,48]. Although there are conflicting results regarding the role of Axl for mammarenavirus cell entry [31,49], Shimojima et al. showed that deletion of the first immunoglobulin-like domain of Axl abolished LASV cell entry [32]. Morizono et al. showed that the presence of Gas6 enhanced LASV infection [33]. A crystal structure of the LG domains from Gas6 in complex with Axl shows the interaction is mediated via an anti-parallel β-zipper and buries a total surface area of ~1100 A^2^ [50]. The LG domains of laminin-α2 can bind matriglycan through the chelation of calcium via two aspartate residues [51] (Figure 1). There is no evidence that the Gas6 LG domains bind matriglycan like those of laminin-α2 [52]. A more likely scenario is that LASV binds cells that express matriglycan but is only internalized in cells which co-express the Axl phagocytic machinery.

Functionally, deletion or substitution of the ATP binding site (K567M), or phosphotyrosine site (Y821F), of Axl cytoplasmic domains prevents LASV entry, suggesting that Axl tyrosine autophosphorylation is essential for LASV entry [35]. This research suggests that cells co-expressing α-dystroglycan modified with matriglycan, Gas6, and Axl or Tyro3 are highly susceptible to LASV entry, whereas non-phagocytic cells such as myocytes, although they express plenty of matriglycan and can bind virions, are incapable of engulfing LASV particles via apoptotic mimicry [34,47,48].

## 3. Subunit GP1 of the Viral Spike Glycoprotein Binds Matriglycan at the Interface Formed upon Trimerization

**Figure 2:**
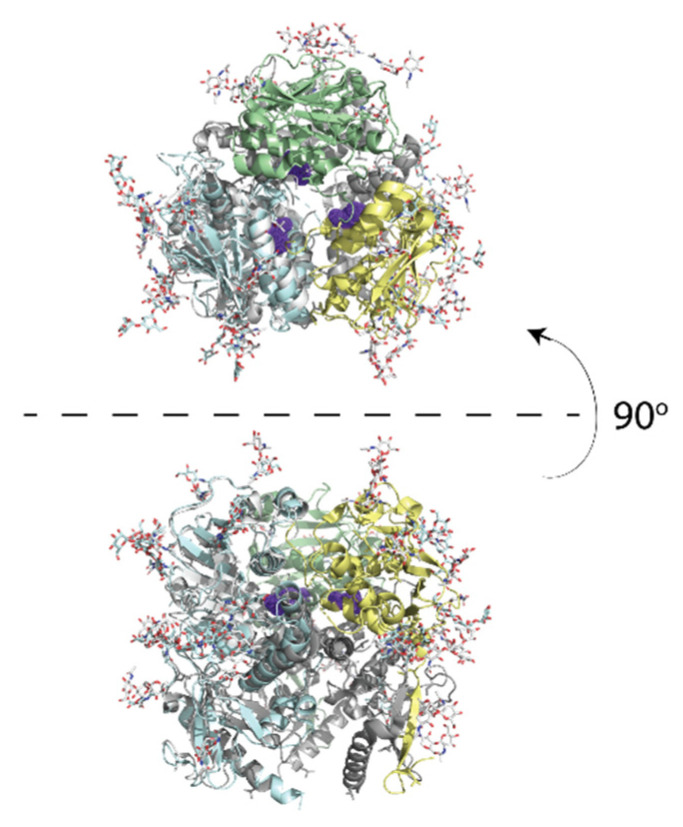
The crystal structure of LASV GP1 trimer is show in cartoon (PDB ID: 5vk2). Leucine-260 is shown in purple on the LCMV trimer. The upper panel shows the top view; the lower panel shows a side view.

Unlike the calcium chelating mode in which LG domains bind matriglycan [4,51], GP1 from LASV and LCMV, which share ~50% sequence identity, likely bind matriglycan using a region formed at the central surface of the trimer. This deduction comes from the fact that the F260 [L/V/I] substitution in the LCMV GP1 increases the affinity for matriglycan by 2–2.5 orders of magnitude and promotes persistent infection in mice [3]. The residues surrounding leucine-260 are essential for viral fusion [53]. The equivalent leucine residue in the trimeric pre-fusion structure of LASV GP1 also maps to their central interface [54], which hints at the location of matriglycan binding (Figure 2).

## 4. Hypothetical Mechanism of Hemorrhage 

LASV-infected cells appear to downregulate matriglycan polymerization [55]. The simplest mechanism might be that the spike protein encounters matriglycan that is nascently polymerized on α-dystroglycan in the Golgi as it traverses the secretory pathway. The incidental exposure of GP1 could disrupt and decrease matriglycan polymerization by LARGE1, which might explain the complex formation and self-limiting infection [55]. A decrease in matriglycan expression has been linked to membrane fragility and treatment of muscle cells with inactivated LCMV interferes with membrane integrity [56]. Patients with high viral load can succumb to hemorrhage, although the mechanism of hemorrhage remains unclear. A physical hypothesis is that compromised cell membranes of blood vessels contribute to hemorrhage. A biochemical hypothesis is that interference with vitamin K-dependent protein-S (ProS1), which is another TAM co-receptor, such as Gas6, acts in complex with ProtC to degrade blood clotting factors Va and VII. Interestingly, ProS1 shares domain architecture and 40% sequence identity with Gas6. Moreover, platelets, which express TAMs, are activated by Gas6 [57]. Perturbation of the ProS1-dependent blood coagulation mechanism eventually causes TAM triple-negative mice to hemorrhage [58]. Interference with platelet function may inhibit coagulation and can cause hemorrhage. Both of these physical and biochemical hypotheses remain to be tested experimentally.

## 5. Conclusions and Remarks

LASV binds host cells via its high-affinity interaction with matriglycan, a repeating disaccharide that is synthesized post-translationally on α-dystroglycan by LARGE1. The spike protein likely binds matriglycan in the central interface formed by trimerization of GP1 monomers. Although virus binding and entry appear to be independent, the former increases the probability of the latter. Viral entry is determined additionally by cell-type-specific factors [17] such as the tyrosine kinase Axl and cell surface lectins. Although studies conflict, many other viruses enter cells using Gas6-Axl mediated apoptotic mimicry [33,44,57,59,60], and this may also explain the cell specificity of the virus. The mechanism of hepatocyte infection and hemorrhage may have to do with parallel tyrosine kinase and LG-domain containing receptors in the liver, such as hepatocyte growth factor receptor (HGFR), and remains to be tested. A decrease in the polymerization of matriglycan may be due to GP1 interacting with the nascent matriglycan polymer on LARGE1-dystroglycan enzyme-substrate complex in the Golgi, which coincidentally prevents further virus entry. Hemorrhage might be the side-effect of conscripting ProS to bind viral glycoprotein resulting in poor blood clotting. We can honor Dr Kunz’ legacy by building on his invaluable work to further explore these unanswered questions.

## Data Availability

All data generated or analyzed during this study are included in this published article.
